# Supplementary material for: Severe COVID-19 infection is associated with aberrant cytokine production by infected lung epithelial cells rather than by systemic immune dysfunction
Source: Res Sq. 2021 Nov 24:rs.3.rs-1083825. Preprint. [Version 1] doi: 10.21203/rs.3.rs-1083825/v1 (PMC8629200; doi:10.21203/rs.3.rs-1083825/v1)
Supplement: 1 [file NIHPPrs1083825v1-supplement-1.pdf]

Supplementary Table 1: Patients excluded from immune analysis

**Reasons for exclusion of patients from immune analyses:**
Trauma/surgery during admission for SARS-CoV-2
Systemic bacterial infection or bacteremia (or high clinical suspicion thereof)
Active malignancy
Biologic / Immune Suppressant use within the last 30 days >= 7.5 mg of prednisone (or within last 6 months for long-acting monoclonal antibodies). Samples prior to receipt of steroids were eligible for inclusion.
Chemotherapy or Immunotherapy within the last 6 months
Sickle Cell Disease with crisis within the last 30 days
Solid organ transplant
Concurrent pneumothorax
Saddle pulmonary embolism with hemodynamic compromise
Cardiogenic shock in a patient with end stage heart failure; out of hospital cardiac arrest
Delivered of a pregnancy during admission for SARS-CoV-2

Supplementary Table 2: Demographic characteristics of patient cohorts

	Healthy donor	Mild	Moderate	Severe	Total SARS-CoV-2 positive cohort	p-value (mild vs severe)
Number of Patients	22	69	30	29	128	
Age; average in years, (range)	44.9 (25-67)	53.3 (21->90)	53.4 (20-79)	61.6 (35->90)	55.2 (20->90)	0.025
Male Sex n (%)	12 (54.5)	25 (36.2)	15 (50)	22 (75.9)	62 (48.4)	7.38 E-04
**Race**						
Black/African-American n (%)	1 (4.5)	40 (58)	25 (83.3)	21 (72.4)	87 (68)	0.254
White n (%)	16 (72.7)	11 (15.9)	1 (3.3)	4 (13.8)	32 (25)	1
Hispanic or Latino n (%)	3 (13.6)	4 (5.8)	3 (10)	3 (10.3)	13 (10.2)	0.419
Asian/Mideast Indian n (%)	0 (0)	3 (4.3)	0 (0)	0 (0)	3 (2.3)	0.553
Did not disclose/unknown n (%)	2 (9.1)	7 (10.1)	1 (3.3)	1 (3.4)	11 (8.6)	0.43
More than one Race n (%)	0 (0)	4 (5.8)	0 (0)	0 (0)	4 (3.1)	0.316
Treated outpatient or discharged from ED n (%)	---	27 (39.1)	0 (0)	0 (0)	27 (21.1)	1.31 E-05
Mean LOS for hospitalized patients; average in days (range)	---	5.8 (0-27)	8.9 (3-25)	16.4 (5-56)	9.8 (0-56)	1.01 E-07
Died During Index Admission n (%)	---	0 (0)	0 (0)	3 (10.3)	3 (2.3)	
**Comorbidities**						
CAD or PAD n (%)	---	7 (10.1)	1 (3.3)	8 (27.6)	16 (12.5)	0.409
COPD or Asthma n (%)	---	16 (23.2)	7 (23.3)	7 (24.1)	30 (23.4)	0.256
Diabetes n (%)	---	19 (27.5)	10 (33.3)	18 (62.1)	47 (36.7)	0.337
ESRD n (%)	---	2 (2.9)	3 (10)	3 (10.3)	8 (6.3)	0.649
HTN n (%)	---	37 (53.6)	15 (50)	22 (75.9)	74 (57.8)	0.389
**SARS-CoV-2 treatments**						

Azithromycin n (%)	---	14 (20.3)	9 (30)	14 (48.3)	37 (28.9)	0.626
Lopinavir/Ritonavir n (%)	---	2 (2.9)	2 (6.7)	0 (0)	4 (3.1)	0.498
Remdesivir n (%)	---	14 (20.3)	15 (50)	14 (48.3)	43 (33.6)	0.626
Steroid n (%)	---	0 (0)	0 (0)	1 (3.4)	1 (0.8)	0.451
Tocilizumab* n (%)<br>*Excluded from cytokines	---	9 (13)	5 (16.7)	3 (10.3)	17 (13.3)	0.203

Significance was calculated using Fisher's Exact Test

Supplementary Table 3. Demographic characteristics of COVID-19 autopsy cases. Abbreviations used: AAA - Abdominal aortic aneurysm; CABG - coronary artery bypass graft; CAD - coronary artery disease; CKD - chronic kidney disease; CVA - cerebrovascular accident; DM - diabetes mellitus; HTN - hypertension; IDDM - insulin dependent diabetes mellitus; pHTN - pulmonary hypertension; OA - osteoarthritis; SLE - systemic lupus erythematosus.

Patient ID	Sex	Age	Race/ethnicity	Comorbidities	Days hospitalized prior to death
1	M	36	AA	Obesity, IDDM	9
2	F	50	Hispanic	HTN, pHTN, COPD, CAD, CVA	12
3	F	82	AA	Alcohol abuse, OA, nursing home resident	13
4	F	68	AA	CVA, SLE, C. difficile, Osteomyelitis	4
5	F	84	AA	CABG, IDDM, dementia, breast cancer	4
6	M	84	AA	CVA, AAA, prostate cancer, nursing home resident	1
7	M	40	AA	Obesity, HTN, IDDM, CAD	21
8	F	77	AA	HTN, DM, dementia	15
9	F	80	Caucasian	HTN, IDDM, COPD, CKD	1
10	F	78	AA	HTN	4

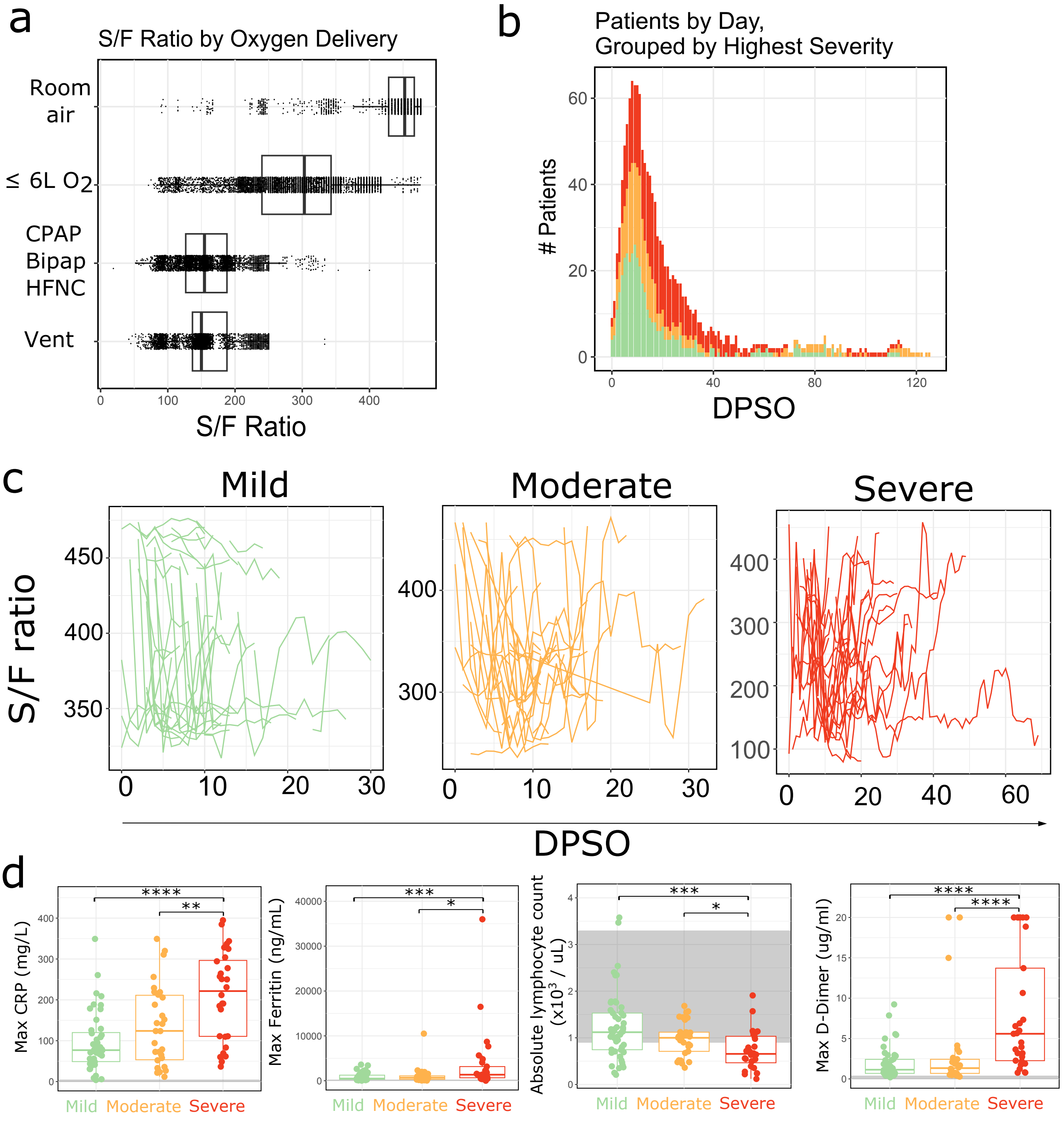

Supplemental Fig 1: Clinical features of study cohort (a) The relationship between S/F ratio and oxygen delivery is depicted. All values during initial admission for all patients (n = 126) are included. Each dot represents an individual S/F ratio, and multiple ratios per patient per day are plotted. This includes individual values with the SpO2 < 88% which preceded an increase in the patient's clinical oxygenation requirement. (b) Stacked histogram showing number of patients hospitalized versus days post symptom onset; each patient is represented multiple times based on duration of hospitalization. Disease severity is indicated by color (mild - green, moderate - orange, severe - red). (c) Mean daily S/F ratio is shown for mild, moderate and severe patients over time, with data from the same patients connected by lines. (d) Maximum CRP, Ferritin, D-Dimer and nadir of absolute lymphocyte count are shown for mild, moderate, and severe patients. Each dot represents the maximum or nadir value for each patient over the course of their initial hospitalization. The grey line indicates the maximum normal value. The grey block background indicates the normal reference range. Significance was determined by two-sided Mann Whitney Wilcoxon test and p-values are indicated by asterisks (*, $p \le 0.05$; **, $p \le 0.01$; ***, $p \le 0.001$, ****, $p \le 0.0001$).

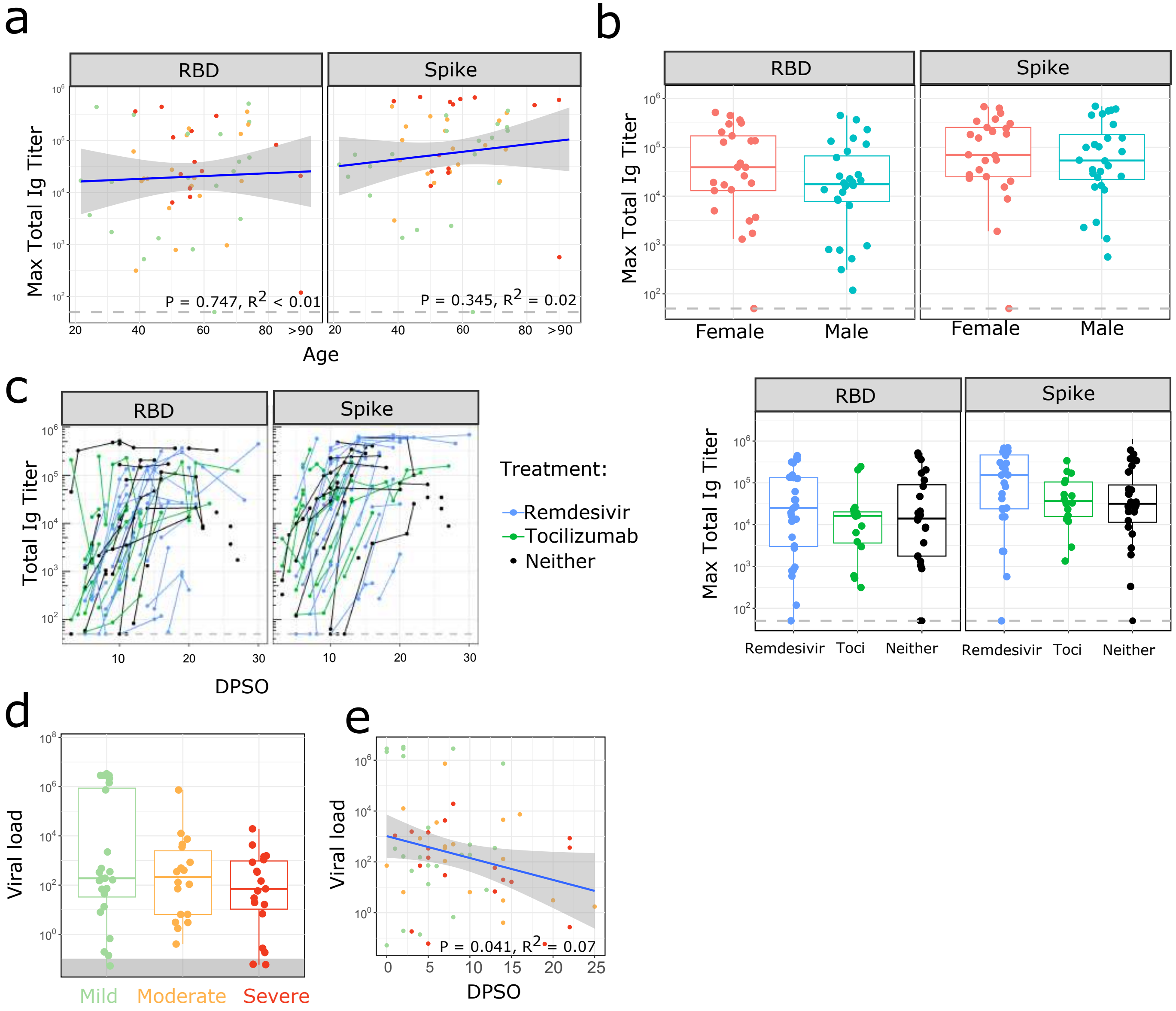

Supplemental Fig 2: Antibody response does not differ based on age, gender, or treatment with remdesivir or tocilizumab (a) Maximum serum SARS-CoV-2 total Ig antibody levels (RBD and Spike) between D10-30 in PCR+ patients (n = 53) are shown by age. Disease severity indicated by color (green = mild, orange = moderate, red = severe). (b) Maximum serum SARS-CoV-2 total Ig antibody levels (RBD and Spike) between D10-30 in PCR+ patients are shown for females (F, n=25) and males (M, n=28). (c) Serum SARS-CoV-2 total Ig antibody levels (RBD and Spike) in PCR+ patients receiving remdesivir (blue, n=28), tocilizumab (green, n=16), or neither (black, n=24). Antibody levels over time shown in the left panel, and maximum titer per patient shown in the right panel. (d-e) Viral titers were obtained by ddPCR on leftover viral transport medium. Disease severity indicated by color (green = mild, n=24; orange = moderate, n=18; red = severe disease, n=19) patients. In d), the grey shaded area denotes negative results. (e) Linear correlation between day post symptom onset and viral load measured by ddPCR, disease severity is indicated by dot color. (a,e) Shaded areas represent 95% confidence interval. (b,c,d) Groups were compared with two-sided Mann Whitney Wilcoxon test and no significant differences were seen.

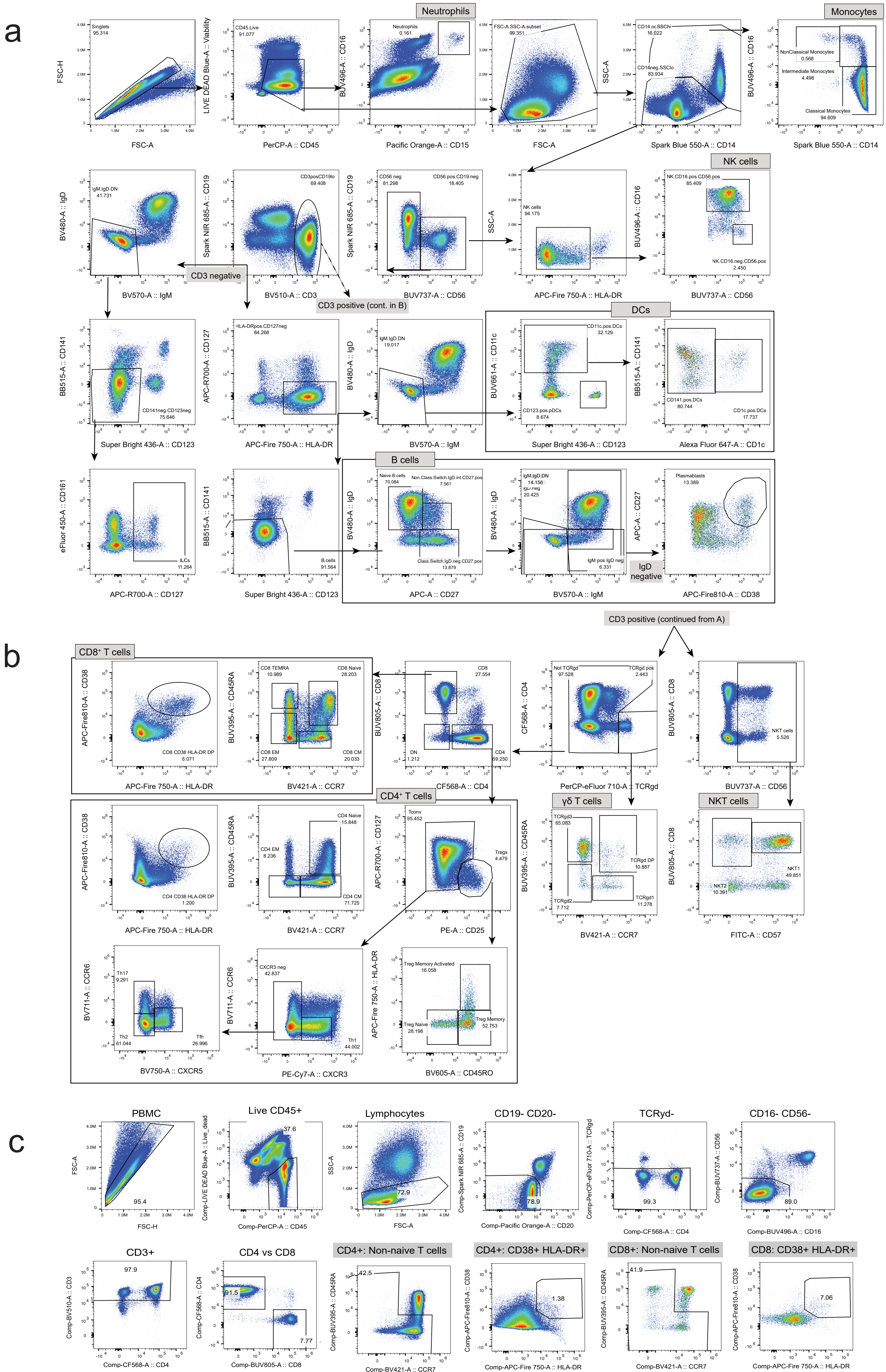

Supplemental Fig. S3

Supplemental Fig 3: Flow cytometry gating strategy. (a) Gating for extracellular flow cytometry for monocytes, dendritic cells, B cells, and NK cells. (b) Gating for extracellular flow cytometry for T cells, ɣδ T cells, and NKT cells. (c) Flow cytometry gating for intracellular cytokine stimulation assays.

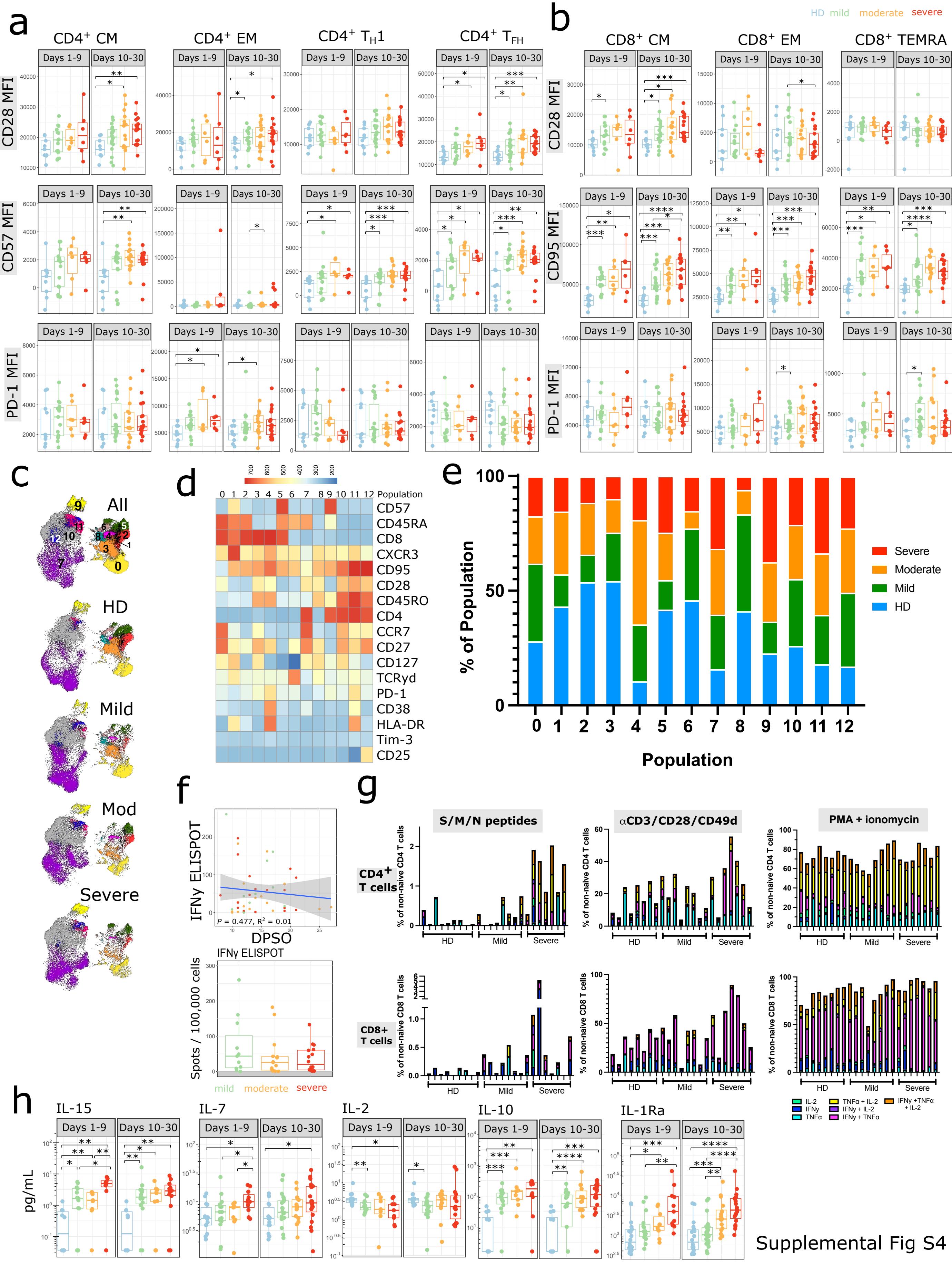

Supplemental Fig S4

Supplemental Fig 4: SARS-CoV-2 infection is associated with increased activation and functionality of CD4+ and CD8+ T cells. (a-b) Boxplots show MFI of indicated markers in each CD4+ (a) or CD8+ (b) T cell population. Where multiple timepoints within the early (D1-9) or late (D10-30) phase per patient were available, the mean was taken and each patient is represented by one dot per time phase. n = 9 for HD (blue), n = 15, 6, 6 for mild (green), moderate (orange), and severe (red) respectively in the early phase, and n = 18, 15, 17 for mild, moderate and severe in the late phase. (c) UMAP projections of live, CD45+ CD3+ cells from samples between D11-17 DPSO (equal numbers of cells sampled from n=7, 7, 7, 7 HD, mild, moderate, and severe patients, respectively) with FlowSOM clusters overlayed. (d) Marker expression in each FlowSOM cluster shown. (e) The percentage of each cluster derived from HD (blue), mild (green), moderate (orange) or severe (red) patients. (f) PBMCs were stimulated with peptides from the S, M, or N proteins in separate wells for 18 hours and IFN-γ production measured by ELISPOT. Response to S, M, and N peptides was summed and normalized per 100,000 cells plated. IFN-γ ELISPOT response shown as a linear correlation with DPSO with disease severity indicated by color with shaded area representing 95% confidence interval (top panel) and as a boxplot by disease severity (bottom panel); n = 10, 17, 18 in mild (green), moderate (orange), and severe (red), respectively. (g) Cytokine production after stimulation with pooled peptides from the S, M and N proteins, αCD3/CD28/CD49d antibodies, or PMA and ionomycin is shown for each individual patient. Background activity in unstimulated wells was subtracted from stimulated wells. The percentage of cells producing various combinations of IFN-γ, TNFα, and IL-2 were reported. HD: n = 9 for SMN stimulation, n = 8 for αCD3/CD28/CD49d and PMA+ionomycin stimulation; mild n = 8; severe n = 7. (h) Maximum cytokine and chemokine levels related to T cell homeostasis are shown during the early phase (days 1-9 from symptom onset) and late phase (days 10-30 from symptom onset) of disease in SARS-CoV-2 infection compared to non-infected healthy controls. Each dot represents maximum value per individual subject during each phase of disease (early phase: mild, n=15; moderate, n=10; severe, n= 11 and late phase: mild, n=23; moderate, n=16; severe, n= 19 and non-infected healthy controls, n=18). (a, b, f, h) Significance was determined by two-sided Mann Whitney Wilcoxon test and p-values are indicated by asterisks (*, $p \le 0.05$; **, $p \le 0.01$; ***, $p \le 0.001$, ****, $p \le 0.0001$).

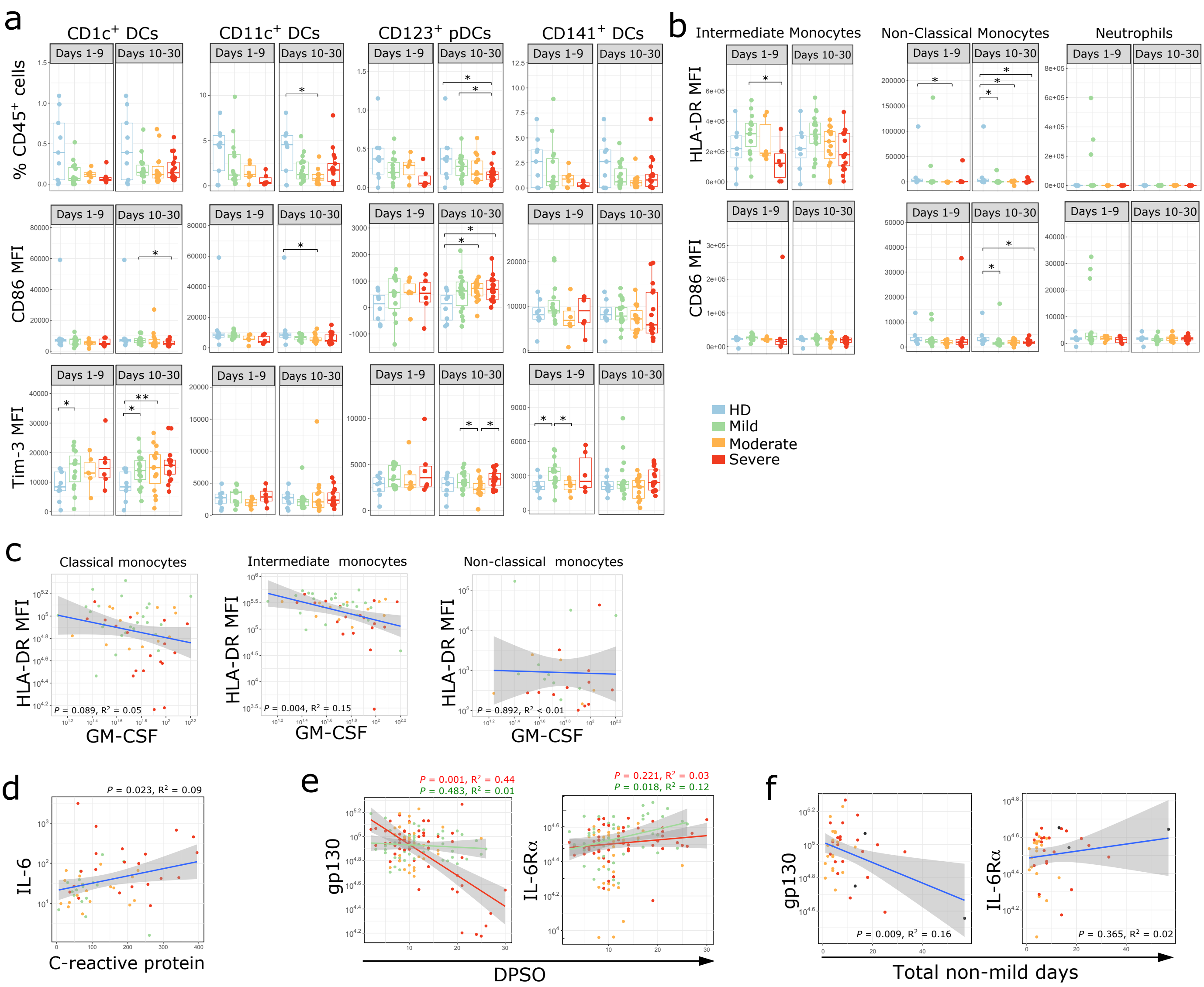

Supplemental Fig 5: Induction of innate immune responses in SARS-CoV-2 infection. (a,b) Immune cell subsets related to the innate immune response are shown. Boxplots show percentages of each cell population out of live CD45+ cells or MFI of indicated markers on dendritic cells, monocytes or neutrophils. Where multiple timepoints within the early (D1-9) or late (D10-30) phase per patient were available, the mean was taken and each patient is represented by one dot per time phase. n = 9 for HD, n = 15, 6, 6 for mild, moderate, and severe respectively in the early phase, and n = 18, 15, 17 for mild, moderate and severe in the late phase. (c) The MFI of HLA-DR on classical, intermediate, and non-classical monocytes is shown as a linear regression with peak serum GM-CSF levels in n = 24, 13, 18 mild (green), moderate (orange), and severe (red) patients respectively. (d) Linear regression is shown for peak serum IL-6 and peak clinical CRP level. Each dot represents the maximum value per individual subject during the disease (mild, n=17; moderate, n=18; severe, n= 21). (e) Linear correlations between peak serum gp130 or Il-6Rα levels versus DPSO in severe (red) and mild (green) patients; disease severity is indicated by color in mild (green, n = 23), moderate (orange, n= 19), and severe (red, n= 33) patients. (f) Peak individual serum levels of gp130 and IL-6Rα are shown as linear correlations with the sum of days each patient spent in moderate or severe S/F status, termed “non-mild days”. Maximal disease severity indicated by color (moderate [orange, n=20]; severe [red, n= 19], or deceased [black, n= 3]). (a, b) Significance was determined by two-side Mann Whitney Wilcoxon test and p-values are indicated by asterisks (*, p $\leq$ 0.05; **, p $\leq$ 0.01; ***, p $\leq$ 0.001, ****, p $\leq$ 0.0001). (c-f) Shaded areas represent 95% confidence interval.

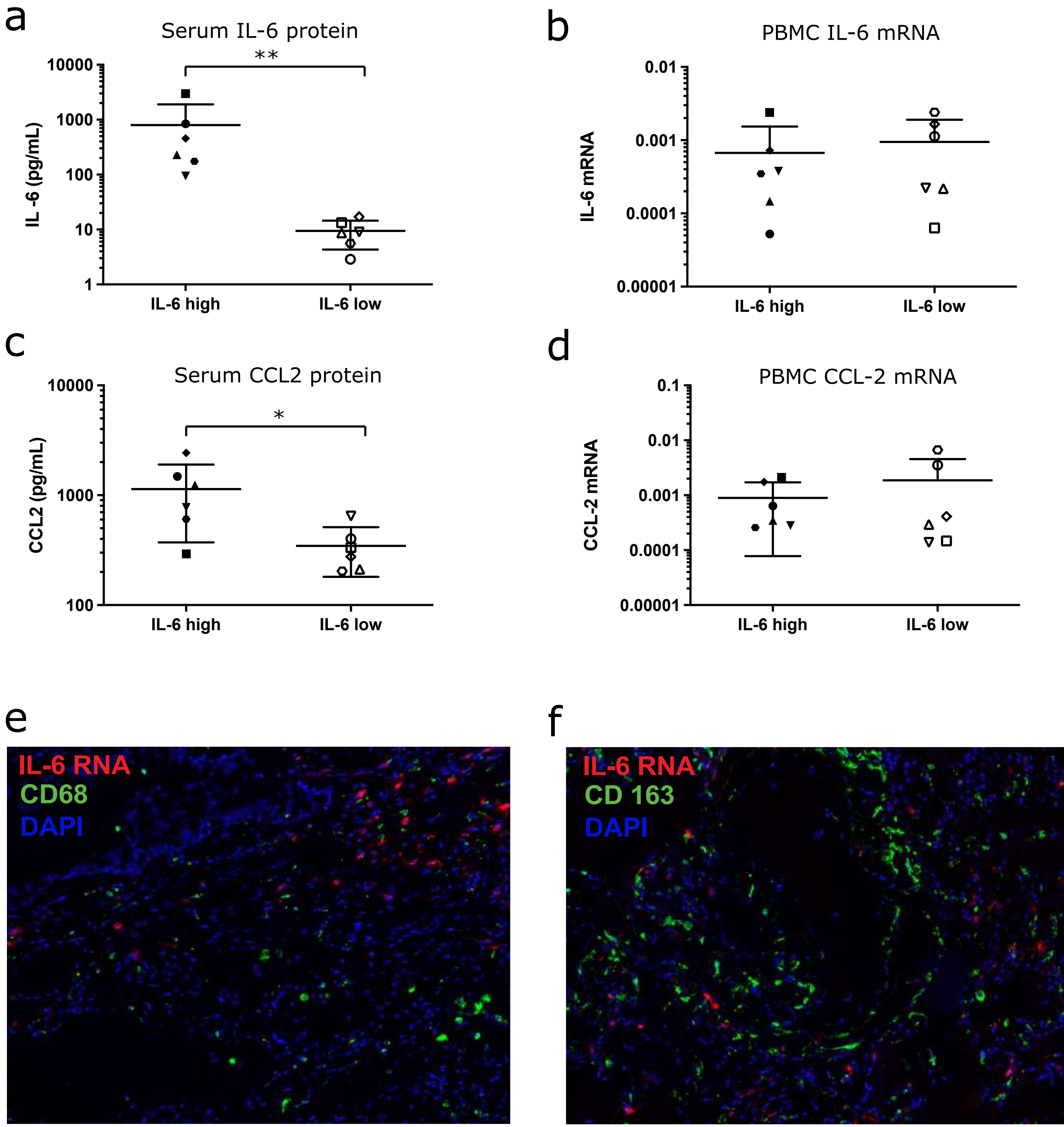

Supplemental Fig 6: IL-6 and CCL2 are not predominantly produced by macrophages. (a-d) RNA was extracted from PBMCs from 6 patients who had either high or low levels of serum IL-6. Serum protein levels of IL-6 (a) and CCL-2 (b) in this cohort are shown. Expression of IL-6 (c) and CCL2 (d) mRNA from PBMCs was evaluated by RT-PCR. Error bars in (a-d) show mean + SD. Significance was determined by two-side Mann Whitney test and p-values are indicated by asterisks (*, $p \leq 0.05$; **, $p \leq 0.01$). (e-f) Representative image showing IL-6 expression outside of CD68+ (e) and CD163+ (f) macrophages. Multispectral images were acquired at 40x magnification. Multiplex immunofluorescence staining was performed for IL-6 RNA (red) and CD68 (panel e, green) or CD168 (panel f, green), and nuclear DAPI counterstain (blue).

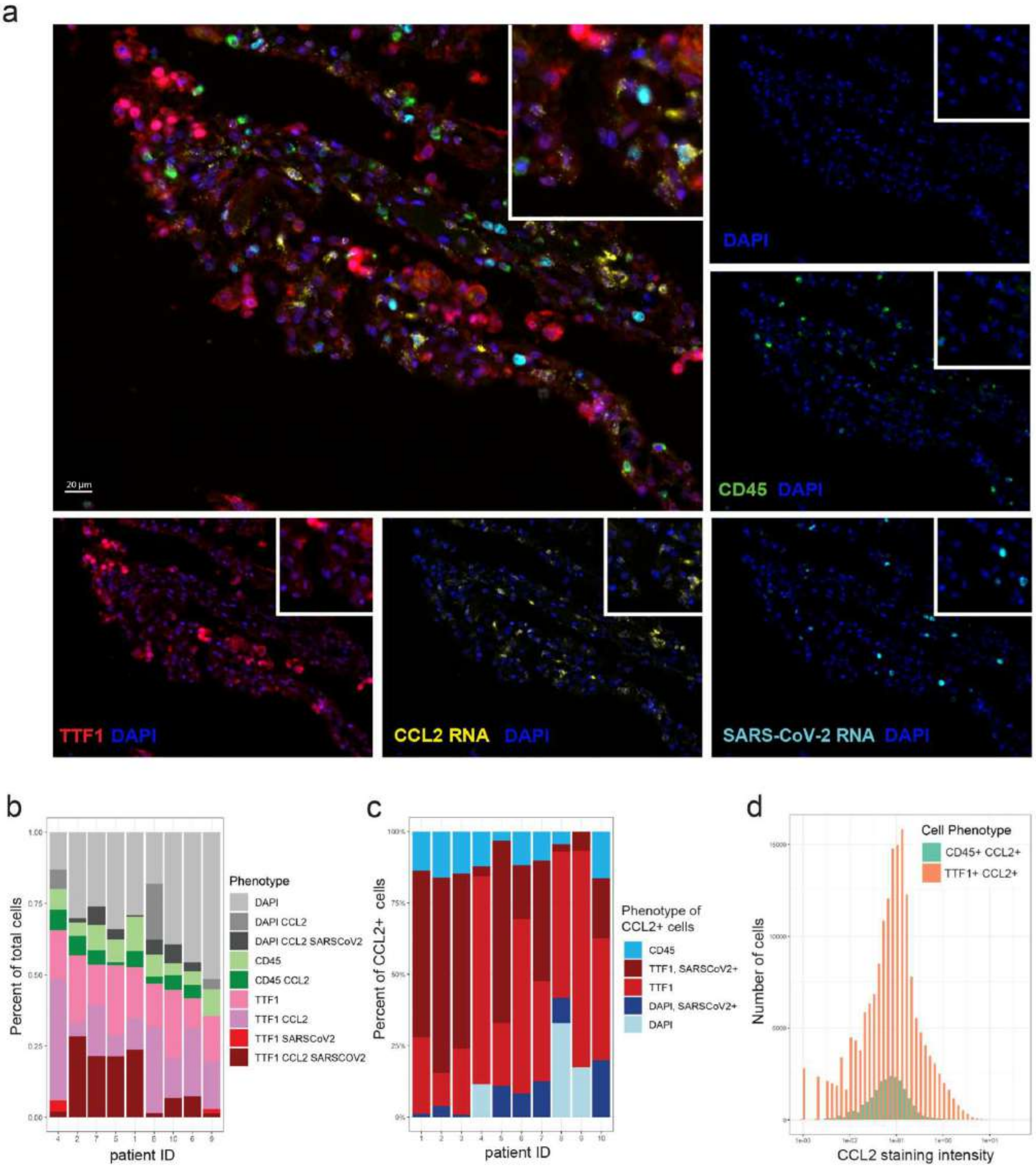

Supplemental Fig 7: Cellular source of CCL2 in autopsy lung tissue in fatal COVID-19. (a) Representative staining for TTF1 (red), CD45 (green), CCL2 RNA (yellow), SARS-CoV-2 RNA (light blue), and nuclear DAPI counterstain (blue); each stain shown separately and merged. Overlaying high-power images showing TTF1+ pneumocytes expressing high levels of CCL2. (b) Bar plots showing the phenotype composition of cell populations in each autopsy lung specimen. (c) Bar plots showing the phenotype composition of CCL2+ cells in each autopsy lung specimen. (d) Histogram displaying the frequency distribution of mean staining intensity for CCL2 between TTF1+ CCL2+ cells (red) versus CD45+ CCL2+ cells (aqua). Cumulative data from all patients shown.
